# Artificial Intelligence and 3D Scanning Laser Combination for Supervision and Fault Diagnostics

**DOI:** 10.3390/s22197649

**Published:** 2022-10-09

**Authors:** Javier Vives, Juan Palací

**Affiliations:** 1 Department of Systems Engineering and Automation, University Polytechnic of Valencia, 46022 Valencia, Spain; 2 Red Engineering Technology Limited, Wolverton, Milton Keynes MK12 5DJ, UK

**Keywords:** deep learning, interferometry, fault diagnosis, RRI, machine learning, condition monitoring

## Abstract

In this work, we combine some of the most relevant artificial intelligence (AI) techniques with a range-resolved interferometry (RRI) instrument applied to the maintenance of a wind turbine. This method of automatic and autonomous learning can identify, monitor, and detect the electrical and mechanical components of wind turbines to predict, detect, and anticipate their degeneration. A scanner laser is used to detect vibrations in two different failure states. Following each working cycle, RRI in-process measurements agree with in-process hand measurements of on-machine micrometers, as well as laser scanning in-process measurements. As a result, the proposed method should be very useful for supervising and diagnosing wind turbine faults in harsh environments. In addition, it will be able to perform in-process measurements at low costs.

## 1. Introduction

Researchers have developed new techniques to maintain wind power infrastructure that have increased wind production by about 60% in the past few years [1]. A wind turbine’s reliability, safety, and profitability can be enhanced through advanced monitoring and fault diagnosis. The maintenance of wind turbines has traditionally relied on fault tree analysis and spectral analysis.

Artificial intelligence (AI) is becoming more popular because of advances in digital and mobile technology. The impact of machine learning has grown even more in these areas because of new hardware and cloud-based solutions [2]. Mechanical or electrical failures are usually the cause of vibration. Gear and bearing failure can also be signaled by vibrations. Bearing wear is primarily due to their rolling elements since their surface position adjusts continuously along with the load. These areas have been impacted even more by machine learning since the introduction of new hardware and cloud-based solutions. Vibrations can also be caused by component failure, cage failure, imbalance, and misalignment in addition to geometric imperfections [3].

It is easier to integrate interferometric techniques (including range-resolved interferometry) into mounting structures since they provide illumination and detection from one point. In monitoring, optical coherence tomography (OCT), an interferometric technique such as range-resolved interferometry (RRI) [4,5,6], is widely used. The maximum working range for OCT can achieve a few millimeters (compared to up to 10 cm for RRI), but can in principle achieve 0.01 mm. OCT systems’ limited operating range prevents self-referencing 3D scanners from being determined, as shown in this study. Besides, typical swept-source OCT systems for monitoring processing applications cost approximately USD 150,000, compromising the low-capital cost-advantage of surveillance and fault detection. With its use of a monolithic laser diode in the telecommunications industry and other less expensive fiber components, RRI’s OCT system compares very favorably with traditional systems, where complex laser sources account for a significant portion of component costs.

Based on AI, machine learning continues to work perfectly [7]. Although this kind of methodology has some limitations, it does have some drawbacks as well. Automatically detecting and categorizing the malfunctioning function of a component is possible using maintenance methodologies. As a result of machine learning, response times are reduced and errors are virtually eliminated, while data management and analysis lead for flexible offshore implementation as well as feedback learning. An AI method must be validated before it can be implemented on a real system without causing costly errors [8]. You can monitor all kinds of failures with AI methodologies by analyzing and preventing them. It is helpful to validate fault diagnosis techniques using prototypes or test benches, in addition to understanding how these systems work, whenever you are developing new techniques, conducting studies, etc. Due to the cost of replacing a broken wind turbine, as well as the loss of energy because they cannot produce energy during peak times, a broken wind turbine can cause considerable losses.

Detecting and diagnosing faults in offshore wind farms is crucial if the machine is to be stopped early if it has a problem, especially in those that have high repair and maintenance costs [9]. Furthermore, maintenance activities must be managed efficiently to decrease downtime and defective product costs. By applying algorithms designed to anticipate and prevent problems, we developed a prototype that detects, supervises, and anticipates failures in contrast to existing systems.

Using vibration analysis and RRI techniques in conjunction with vibration analysis, this paper presents an algorithm for monitoring and diagnosing faults in a prototype wind turbine. The algorithm presented here can detect different types of bearing failures autonomously. Following the literature review, an analysis of the data set and data collection was conducted, followed by a comparison of the results. Several conclusions are drawn at the end of the study.

## 2. Research Methodology

Different methods can be used to diagnose and monitor vibrations in turbine bearings, and each bearing can have different characteristics. Consequently, the bearing characteristics may not always match the fault characteristics. In this study, we demonstrate how machine learning and laser scanning can improve accuracy and predict failure based on vibration measurements taken from another bearing.

### 2.1. Machine Learning

Detecting anomalous behavior and classifying faults are the two main tasks of machine learning techniques for wind turbine fault detection. Furthermore, this technique allows for quick corrective measures in case of a failure or anticipated problem, which improves the system’s performance and security. A supervised machine learning method is the most common, followed by an unsupervised one [10]. When you use supervised learning, you already know the output. The outcome of unsupervised learning is unknown. The process is the same on the way in and out. The only input data required for unsupervised learning are the binary logic that is present in every system, unlike supervised learning. No references are used at all. To apply any type of learning, the data must first be classified. A variety of classification algorithms can be used to solve this problem. The K-Nearest Neighbour (KNN) and Support Vector Machine (SVM) algorithms are two of the most relevant classifiers used in machine learning for supervision, monitoring, and fault diagnosis in a wind turbine [11].

These algorithms combine the functionality of an object with various categories or classes based on the information it provides.

This leads to a two-phase classification process:

- During training, the data must be properly classified, and then the parameters are adjusted to achieve the optimal performance.
- Following training, the algorithm provides an output based on the input data.

### 2.2. Deep Learning

**Figure 1:**
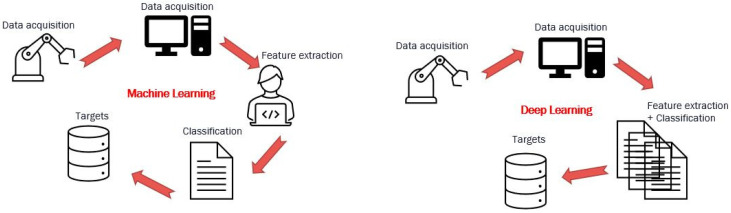
Comparison between machine and deep learning methodology.

The AI algorithms may have difficulty extracting the features of many tasks [12]. Deep learning methods can help overcome these weaknesses in current intelligent fault-diagnosis methods [13] by learning feature hierarchies composed of features from higher levels of the hierarchy [14]. The Venn diagram of the relationship between different AI disciplines is shown in Figure 1. Using deep learning methods, the system can learn complex functions directly by mapping inputs to outputs through automatic learning at multiple levels of abstraction. Deep architectures, composed of multiple nonlinear levels, are required to learn these complicated functions. Deep learning-based methods utilize deep architectures to capture the representation information from natural input signals through nonlinear transformations, and to approximate complex nonlinear functions well. Table 1 shows the main advantages and limitations of some AI classifiers. Table 2 compares the performance of each of the AI classifiers.

Computer vision, audio recognition, natural language processing, as well as fault diagnosis are among the many tasks based on deep learning that have demonstrated state-of-the-art performance in recent years [15,16]. Rotating machinery fault diagnosis has also been carried out using deep learning approaches such as autoencoders, restricted Boltzmann machines (RBMs), and deep belief networks (DBNs).

**Figure 2:**
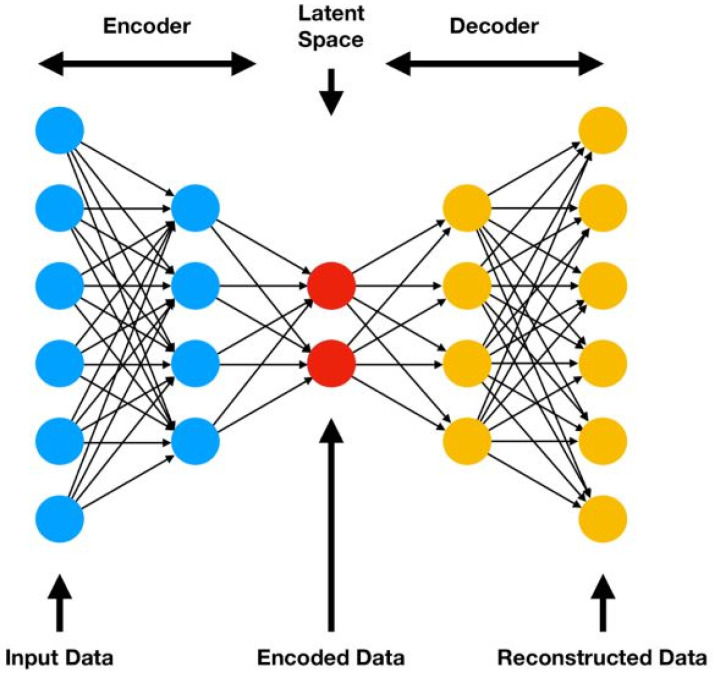
An autoencoder is represented graphically.

A backpropagation-based autoencoder sets the target values to equal the input values, which is an unsupervised learning algorithm, as depicted in Figure 2. Autoencoder NNs are composed of two parts: encoder and decoder. A low-dimensional space is generated from the input data by the encoder, and the input data are reconstructed from the code by the decoder.

A function $h_{W,b}\approx x\;$ is learned by the autoencoder, *W* is the parameter (or weight), and *b* is the bias associated with the connection between two layers. A low-dimensional representation of the original data can be learned since the autoencoder can detect correlations between the data if there are any. A stacked autoencoder is composed of multiple layers of sparse autoencoders, whose outputs are interconnected.

In addition to fault diagnosis, DBN is also a useful tool, which is a series of multiple layers of restricted Boltzmann machines (RBMs); this is a particular type of log-linear Markov Random Field (MRF) called a Boltzmann machine (BM). As a result, its free parameters are linear. Several hidden variables are considered to make it powerful enough to represent complicated relationships. To improve the modulation capacity of the BM, the number of hidden variables (or hidden units) can be increased, such that the deep belief network (DBM) restricts bipartite graphs to those without visible–visible and hidden–hidden connections.

The energy function *E* (*v*,*h*) of an RBM is defined as:

(1) $$E\;\left(v,h\right)=-b^{\prime }-c^{\prime }h-h^{\prime }Wv$$

A hidden unit is connected to a visible unit by a weight *W*. The offsets of the visible layer and the hidden layer are *b* and *c*, respectively. Equation (1) can be translated to the free energy formula:

(2) $$F\left(v\right)=-b^{\prime }v-\underset{i}{\sum }log\underset{h_{i}}{\sum }e^{h_{i}\left(c_{i}+w_{i}v\right)}$$

RBM has a conditionally independent structure between visible and hidden units. Therefore, we can obtain:

(3) $$p\left(h|v\right)=\underset{i}{\prod }p(h_{i}|v)$$

(4) $$p\left(v|h\right)=\underset{j}{\prod }p(v_{j}|h)$$

A deep Boltzmann machine (DBM) can be obtained by increasing the number of hidden layers. To obtain DBN, the Bayes belief network is used at the part closest to the visible layer, while RBM is used at the part away from the visible layer. A deep belief network (DBN) consists of three main principles:

- Unsupervised learning of representations for pre-training;
- Supervised training of each layer on top of previously trained layers;
- Fine-tuning of each layer by supervised training.

### 2.3. Range-Resolved Interferometry (RRI)

By utilizing different processing algorithms, RRI can be tailored to use monolithic laser diodes, a more cost-effective alternative to OCT. In [17,18], RRI’s underlying principles are described. Simple switching techniques are used in this technique, which involve modulating a diode laser with a sinusoidal optical frequency, delivering reflected light from a target (layer surface), and interfering it with the light reflected from a fiber tip used as a reference. An interference signal that is demodulated with a smooth window function gives a sinusoidal signal, in which the frequency indicates how far away the target was from the fiber tip, and the amplitude indicates how bright the reflection was. The center position of sinusoids along the laser beam, relative to the fiber tip position, is recorded for sinusoids whose amplitudes exceed a particular limit.

### 2.4. Implementation of Scanned RRI

**Figure 3:**
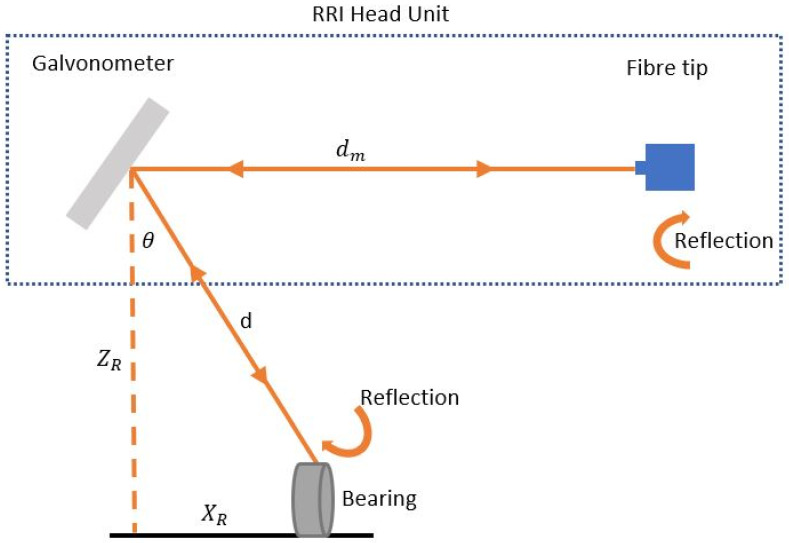
Interferometry for 2D implementation using scanned range-resolved interferometry.

A data rate of 3.2 kHz is provided by the RRI instrument. As shown in Figure 3, the data output is the amplitude of the signal and the angle θ of the galvanometer scan (corresponding to the distance along the laser beam from the fiber tip at each instance). Based on the distance between the galvanometer mirror and the reflection and the galvanometer angle, the RRI head unit can be used to convert the polar coordinates into Cartesian coordinates using the geometrical relations given in Equations (5)–(7):

(5) $$X_{R}=\left(d-d_{m}\right)\cos\theta$$

(6) $$Z_{R}=\left(d-d_{m}\right)\sin\theta$$

(7) $$y_{R}=0$$

Galvanometer angle θ is the distance from the galvanometer to reflection *d*; the distance between the galvanometer and fiber tip $d_{m}$ determines the galvanometer angle. By turning the galvanometer mirror directly back towards the fiber tip, the length $d_{m}$ can be calibrated accurately using the RRI instrument. If the galvanometer mirror angle θ is zero, and the beam is angled vertically, then the equation describes a two-dimensional case (*x*-axis and *z*-axis, across a wall, vertically) in which components on the *y*-axis (along the wall) are absent (i.e., the 2D diagram shown in Figure 3). Thus, the RRI instrument outputs a point-cloud that describes the reflections occurring at specific spatial locations and times $x_{R},\;y_{R},\;z_{R},t_{R}$, each given by an array of values.

## 3. Case Study

The report describes the industrial environment, the components, and the distribution of the laser within the system. A data acquisition method is also explored.

### 3.1. Prototype and Laser Distribution

**Figure 4:**
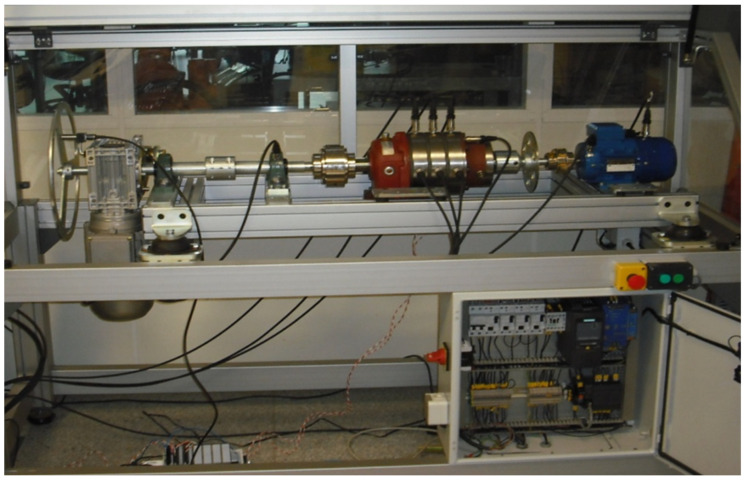
Wind turbine workbench.

Figure 4 shows a small wind turbine prototype showing deterioration and wear on the parts, along with its effects, which can be used to diagnose problems [19]. It allows an easy exchange of parts without having to wait for deterioration to occur, allowing diagnostic techniques to be tested before defects arise. Vibrations are measured in generators, gearboxes, and bearings.

**Figure 5:**
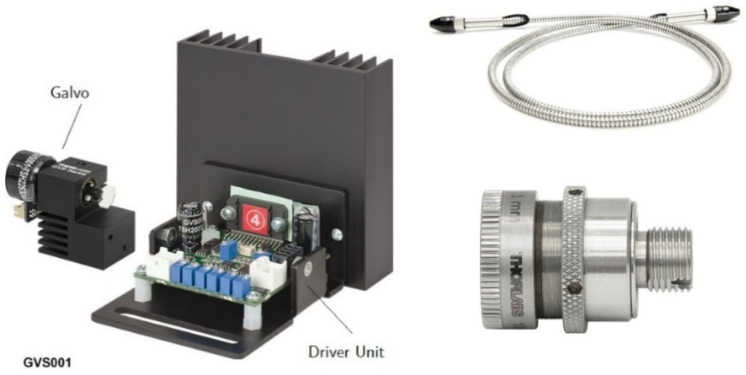
GVS001 Galvo scanner (Thorlabs Inc., Newton, NJ, USA) and the associated drive circuit are shown on the left. An armored fiber cable and an adjustable CFC5-C collimator (Thorlabs Inc., Newton, NJ, USA) are shown on the right.

The scanning laser can be installed in different places of the wind turbine prototype. As the state monitoring techniques and machine design dictate, the scanning laser can be positioned in each stage of the multiplier. To monitor the vibrations caused by the fast shaft coupling to a generator, the laser can be placed in the input bearing. The signal will propagate between the stages and the vibrations will be affected by various failures. On the slow axis, there is also an interesting bearing for measuring the prototype. For some of the damaged bearings, this element can be replaced to determine how the signal behaves after failure, as well as how the signal performs during a normal operation and how the bearing itself deteriorates over time. In Figure 5, we present the galvo laser we used for this study.

### 3.2. Data Collection and Description

Once calibrated using the procedure discussed below, the galvo scan occurred 50 s into the measurement, which corresponds to a 136 mm y-axis position along the bearing. An angular amplitude of 5.8 θ was measured with an angular frequency of 25 Hz and a data rate of 3.2 kHz. On steeper sides, too much light is reflected away from the instrument and the RRI instrument falls below the detected noise level. (For materials with less specular reflection, such as titanium, the RRI instrument will have greatly improved the coverage of the sidewalls because there will be less specular reflection and more scattered light from the angled sidewalls). The RRI instrument measurements are controlled by Python scripts that are started manually and do not automatically sync from measurement to measurement.

## 4. Results and Discussion

Based on the laser scanning data provided in the previous section, the simulation is successfully run. From 0 to 1000 rpm, the prototype is capable of rotating at four different speeds. The medium speed in this case was 200 rpm. Wind turbine failures can be tracked, diagnosed, and prevented with automated learning systems. A 3.2 kHz graphical presentation was generated from an average of 5000 samples generated by a selected scanning laser. Furthermore, automated learning systems can predict wind turbine failures in addition to tracking, preventing, and diagnosing them. A properly trained algorithm can analyze and categorize the data independently after receiving feedback, which allows the algorithm to make a correct prediction. During the simulation, we simulated two states of analysis: good stage and imbalance. A galvo laser scanner was used to acquire the data, followed by filtration and processing, resulting in four phases of analysis.

Due to some external factors found during this experiment, this first stage of filtration and processing the data was crucial. For the filtration, we have set specific ranges/parameters that allow us to pass from the “raw” data to a “clean” data base that we will use in the next steps of the algorithm. Analyzing a signal that is randomly generated will not be stable. To extract patterns from signals of this type, machine learning algorithms must be conditioned appropriately and processed efficiently. In addition to its time variations, the signal is difficult to analyze and learn from. To ensure that the algorithm works correctly, it must first go through this first stage of filtering and conditioning. The invariant characteristics of a signal are read in time by a signal-processing algorithm. The extraction of features is required to determine if a fault or condition is present. Using the number of tests and the number of examples of each problem, the arithmetic mean is calculated. The data set is then reduced to the minimum number of variables necessary to represent the original variables using principal component analysis.

We can also make future decisions based on an understanding of the current state and what is happening, in addition to determining the standard deviation for each of the stipulated failure conditions, such as all machine learning classifiers which are based on mathematical and statistics. There are many states that are dispersed in the data, indicating that most points are close to the average, which is why the model should work.

**Figure 6:**
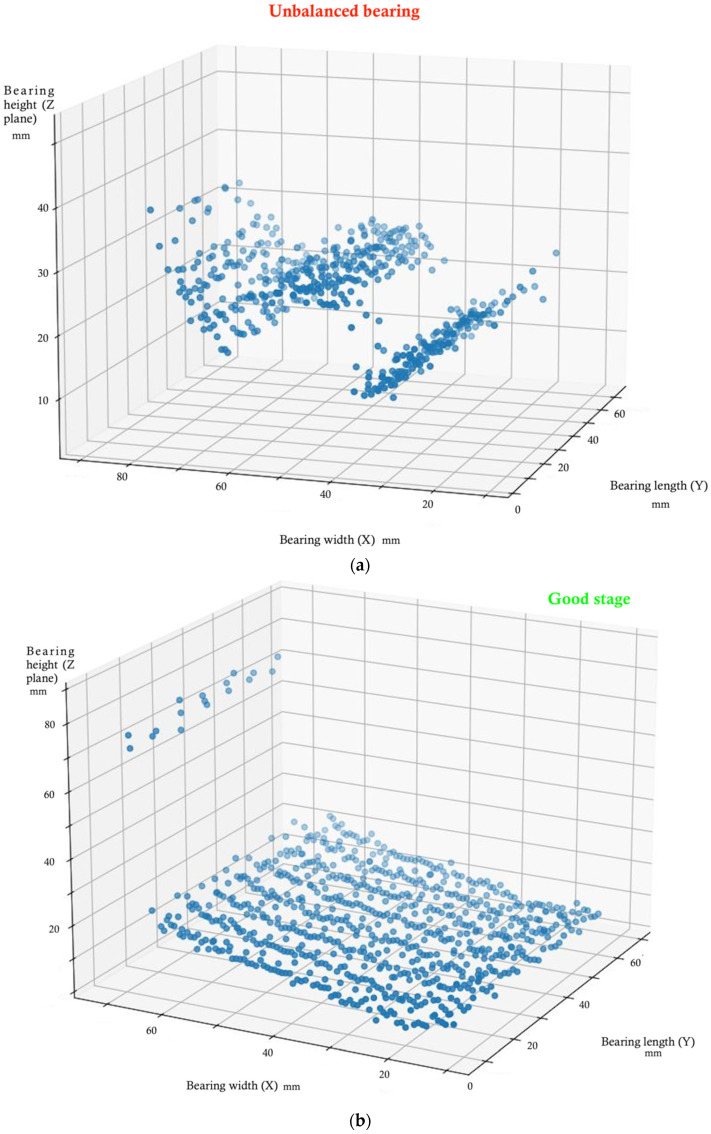
(**a**). Imbalance. Predicted output algorithm; (**b**). Good stage. Predicted output algorithm.

Let us define and explain each one-off simulation. First, the two simulated states start from the mathematical processes explained above. In Figure 6a, an imbalance is presented. We can see that the machine learning algorithm implemented presents a few interesting aspects to define. The plot obtained from Python scrips perfectly represents an imbalance in the wind turbine prototype. We can see that there are some points that the 3D laser scanner post-processor has confused as possible noise. Due to the limits, we have found in this case that the imbalance algorithm is a bit out of date regarding their feedback. Let us explain the results with more detail. The data analyzed have the same shape of a sinusoid wave, which is due to the imbalance. This graph represents a perpendicular plane section of the bearing.

In Figure 6b, a good bearing stage is presented. We can see that the data predicted by the scanning laser are always very stable. In total, 5000 different points are presented. It is true that there are a few wrong predictions where the algorithm confuses the good stage with something unknown. The data analyzed are completely flat because the scanner is not recording any anomalies in the bearing, which means that the bearing works as expected. Such as before, this graph represents a perpendicular plane section of the bearing.

Using the right combination of the AI algorithm and laser scanning software, the data are grouped well in both cases. The stages are classified and analyzed correctly. The algorithm is highly accurate and produces the predicted output with a high degree of similarity regardless of the two failure conditions (imbalance and good stage).

If we compare with the previous studies presented in the introduction, the methodology implemented provides a very good result with the combination of the 3D scanning laser. The simulations and predictions of the two conditions stipulated show that it can be implemented in a real wind turbine. Until now, all studies implemented for supervision and fault diagnoses are using the traditional methods, such as frequency analysis or vibration analysis. These results are stuck and are very limited by the parameters that can monitor, predict, and anticipate future breakdowns; the methodologies presented (3D scanner and machine learning) are the best combination for monitoring in real time.

AI methodologies combined with 3D scanner lasers are, therefore, considered to have a lot of potential for future development in the maintenance sector, as the variables, simulations, and results we considered work well with our wind turbine prototype, enabling us to predict the failures of the prototype with a high degree of accuracy.

## 5. Conclusions

Acquiring and classifying data play critical roles in AI’s success and proper operation. Wind turbine faults are easier to detect, monitor, and diagnose due to the combination of scanning lasers and machine learning systems. Here, both technologies are investigated and combined to diagnose and prevent bearing failures in wind turbines through vibration analysis. In-process supervision and failure diagnosis can be made easier with the RRI instrument. The results obtained during and after processing did not differ significantly in quality. RRI measurements using a galvanometer provide good coverage of the bearing up to a scanning angle of 4 degrees.

A combination of AI and scanning lasers can diagnose bearing faults, which is very suitable for this type of study because of its robustness, high accuracy, and high processing speed. As a result, the methodology identifies and prevents the possible breakdown of other mechanical components of wind turbine prototypes, allowing it to be applied to other mechanical components of wind turbine prototypes. With this prototype, fault diagnosis and supervision techniques can be studied, developed, and validated with the possibility of replacing defective or worn parts with other components. The prototype wind turbines are used to test the diagnostic algorithms that are to be installed in high performance wind turbines. This allows for cost and time savings, as well as the ability to verify, adjust, and correct the algorithms.

## Figures and Tables

**Table 1:** Artificial intelligence: advantages and limitations.

Algorithm	Advantages	Limitations
*k-NN*	Easy to implement. Can be used for both classification and regression.	Big reckoning. It needs a lot of storage space. The selection of k influences the classification.
*SVM*	High sorting accuracy. Can deal with high dimensional features.	Low efficiency for big data. No physical meaning.
*Deep learning*	Automatic fault recognition and learning features. It does not need the function extractor.	Large sample needs. No physical meaning. A lot of training.

**Table 2:** Performance Comparison.

	*SVM*	*k-NN*	*Deep Learning*
Sorting speed	****	*	**
Overall accuracy	****	**	****
Noise robustness	**	*	****
Overfitting	**	***	***
Robustness to parameters	*	***	**
Physical explanation	*	***	*

## Data Availability

Not applicable.

## References

[1] Yang B., Liu R., Chen X. (2018). Sparse time-frequency representation for incipient fault diagnosis of wind turbine drive train. IEEE Trans. Instrum. Meas..

[2] Dey S., Saha S., Singh A.K., McDonald-Maier K. (2022). SmartNoshWaste: Using Blockchain, Machine Learning, Cloud Computing and QR Code to Reduce Food Waste in Decentralized Web 3.0 Enabled Smart Cities. Smart Cities.

[3] Das D., Das A.K., Pratihar D., Roy G. (2020). Prediction of residual stress in electron beam welding of stainless steel from process parameters and natural frequency of vibrations using machine-learning algorithms. Proc. Inst. Mech. Eng. Part C J. Mech. Eng. Sci..

[4] Pankratz H.G., Sultan M., Abdelmohsen K., Sauck W.A., Alsefry S., Alharbi H., Emil M.K., Gebremichael E., Asaeidi A., Alshehri F. (2021). Use of Geophysical and Radar Interferometric Techniques to Monitor Land Deformation Associated with the Jazan Salt Diapir, Jazan city, Saudi Arabia. Surv. Geophys..

[5] Pawluszek-Filipiak K., Borkowski A. (2020). Monitoring mining-induced subsidence by integrating differential radar interferometry and persistent scatterer techniques. Eur. J. Remote Sens..

[6] Ye Y., Li X., Xu Y., Ding L., Su Z., Huang Y., Guo X., Zhang D. (2022). Simultaneous 3D measurement for infrared chips with speckle interferometry. Opt. Laser Technol..

[7] Cai B., Wang Z., Zhu H., Liu Y., Hao K., Yang Z., Ren Y., Feng Q., Liu Z. (2021). Artificial Intelligence Enhanced Two-Stage Hybrid Fault Prognosis Methodology of PMSM. IEEE Trans. Ind. Inform..

[8] Karasev P.A., Sokolov V.V., Sharyapov R.A. (2021). Thomas Theorem, methodology of Information Operations and applications of Artificial Intelligence. Int. J. Open Inf. Technol..

[9] Ren Z., Verma A.S., Li Y., Teuwen J.J., Jiang Z. (2021). Offshore wind turbine operations and maintenance: A state-of-the-art review. Renew. Sustain. Energy Rev..

[10] Mohammadnazar A., Arvin R., Khattak A.J. (2020). Classifying travelers’ driving style using basic safety messages generated by connected vehicles: Application of unsupervised machine learning. Transp. Res. Part C Emerg. Technol..

[11] Demidova L. (2021). Two-Stage Hybrid Data Classifiers Based on SVM and kNN Algorithms. Symmetry.

[12] Dong S., Wang P., Abbas K. (2021). A survey on deep learning and its applications. Comput. Sci. Rev..

[13] Xie S., Yu Z., Lv Z. (2021). Multi-Disease Prediction Based on Deep Learning: A Survey. Comput. Model. Eng. Sci..

[14] Ranganathan G. (2021). A study to find facts behind preprocessing on deep learning algorithms. J. Innov. Image Process. (JIIP).

[15] Medina R., Vasseur R., Serbyn M. (2021). Entanglement transitions from restricted Boltzmann machines. Phys. Rev. B.

[16] Nomura Y. (2021). Helping restricted Boltzmann machines with quantum-state representation by restoring symmetry. J. Phys. Condens. Matter.

[17] Jian M., Lu Z., Chen V.C. Drone detection and tracking based on phase-interferometric Doppler radar. Proceedings of the 2018 IEEE Radar Conference (RadarConf18).

[18] Kissinger T., Chehura E., Staines S.E., James S.W., Tatam R.P. (2017). Dynamic Fiber-Optic Shape Sensing Using Fiber Segment Interferometry. J. Light. Technol..

[19] Yao S., Griffith D.T., Chetan M., Bay C.J., Damiani R., Kaminski M., Loth E. (2020). A gravo-aeroelastically scaled wind turbine rotor at field-prototype scale with strict structural requirements. Renew. Energy.

